# Renal Technician Programs at US Postsecondary Institutions

**DOI:** 10.1016/j.xkme.2025.101224

**Published:** 2025-12-19

**Authors:** Laura C. Plantinga, Danilo Concepcion, Megan Urbanski, Christin Iroegbu, Delphine Tuot, Bernard G. Jaar

**Affiliations:** 1 Division of Nephrology, Department of Medicine, School of Medicine, University of California San Francisco, San Francisco, CA; 2 Philip R. Lee Institute for Health Policy Studies, University of California San Francisco, San Francisco, CA; 3 Independent Dialysis Consultant, Chino Hills, CA; 4 Department of Nursing, Towson University, Baltimore, MD; 5 Department of Surgery and Health Services Research Center, Emory University, Atlanta, GA; 6 Departments of Medicine and Epidemiology and Welch Center for Prevention, Epidemiology, and Clinical Research, Johns Hopkins University, Baltimore, MD

To the Editor:

Over 40,000 dialysis patient care technicians (PCTs) provide complex frontline care for US patients receiving hemodialysis. Responsibilities include initiating and discontinuing dialysis treatment; operating, maintaining, and disinfecting hemodialysis machines; accessing and monitoring fistulae, grafts, and catheters; and monitoring vital signs before, during, and after hemodialysis.^1^ Nationally, dialysis PCTs are required to have a high school degree or equivalent and to be certified; additional state-level requirements (eg, California) may also apply.^2^^,^^3^ However, there is little transparency in, or oversight of, the training for this certification. Although the Conditions for Coverage specify topics to be covered in these programs, the actual content, duration, modality, and intensity of the classroom training may vary considerably, with some programs offered by employers and others offered at community colleges or vocational/technical schools.^2^ A standardized, accredited classroom curriculum for dialysis PCTs, such as that for respiratory therapists, would support consistent training and promote competency across institutions and could be at least partially implemented through US postsecondary institutions.^4^ To better understand the current role and capacity of these institutions in US dialysis PCT training, we explored the availability of classroom dialysis PCT training programs offered by US postsecondary schools and the students completing these programs.

Integrated Postsecondary Education Data System (IPEDS) data (through 2023/2024) are derived from annual National Center for Education Statistics surveys of US postsecondary institutions that participate in federal student financial aid programs. We identified programs using the Classification of Instructional Programs (CIP) code 51.1011 (“Renal/Dialysis Technologist/Technician”). Estimated US PCT FTEs and prevalent counts of end-stage kidney disease patients (through 2022) were obtained from aggregated data provided by the United States Renal Data System.^1^ This study did not meet the criteria for human subjects research set by the University of California San Francisco Institutional Review Board and did not require informed consent. We used descriptive statistics (Stata v. 19.5, College Station, TX) to describe the programs and graduates.

**Figure 1:**
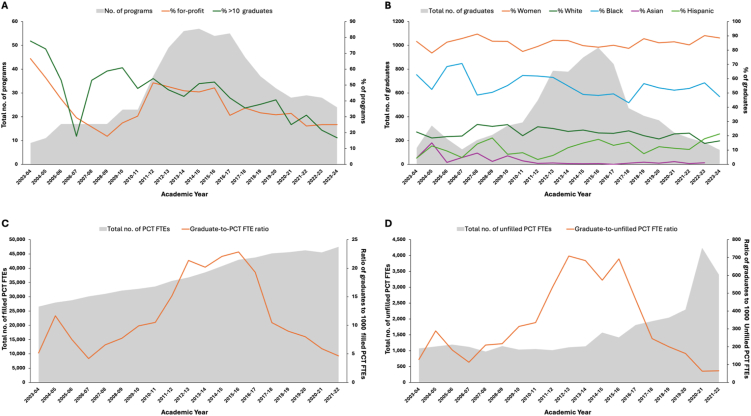
Total number of renal technician programs (A) and total number of awards conferred (B) at US postsecondary schools and ratios of graduates to total numbers of filled (C) and unfilled/open (D) PCT FTEs, per academic year. Note: Aggregated data from the United States Renal Data System on PCT FTEs are currently available through 2022. Abbreviations: FTE, full-time equivalent (estimated as full-time positions + 0.5 × part-time positions); PCT, patient care technician.

**Figure 2:**
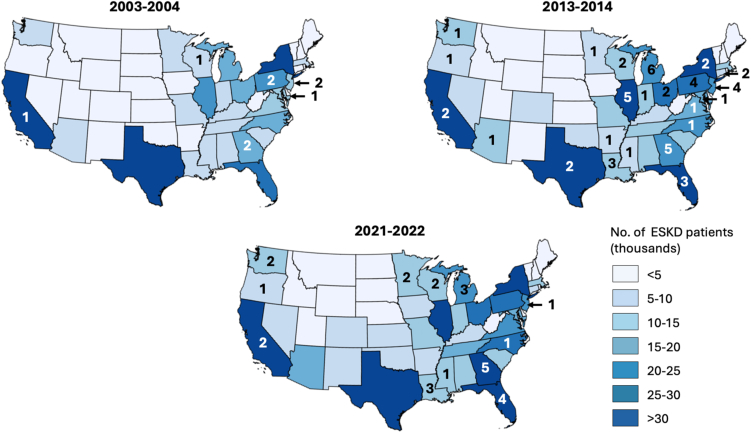
Maps showing the total number of prevalent end-stage kidney disease patients (shading) and number of renal technician programs at postsecondary schools (numbers) in the 2003-2004, 2013-2014, and 2021-2022 academic years, by US state. Note: United States Renal Data System data are available through 2022. There were no programs in Alaska or Hawaii in any time period observed. Abbreviation: ESKD, end-stage kidney disease.

The number of US postsecondary renal technician programs was 9 in 2003-2004, 56 in 2013-2014, and 24 in 2023-2024 (Fig 1A). In the same years, the percentages of programs at for-profit institutions were 66.7%, 46.4%, and 25.0%, respectively, whereas the percentages of programs with ≥10 graduates were 77.8%, 42.9%, and 16.7%, respectively. The numbers of graduates were 137 in 2003-2004, 778 in 2013-2014, and 122 in 2023-2024 (Fig 1B). Although the percentages of graduates who were women (88.5% in 2023-24) or White (16.4% in 2023-2024) remained steady over the study period, the percentages who were Black (62.8% to 47.5%) and Asian (4.4% to 0.8%) declined, and the percentage who were Hispanic increased (4.4% to 21.3%). The ratios of graduates to currently working US PCT FTEs were 5.2/1,000 in 2003-2004, 21.4/1,000 in 2012-2013, and 4.6/1,000 in 2021-2022 (Fig 1C). In the same years, the ratios of graduates to open US PCT FTEs were 128.0/1,000, 708.7/1,000, and 64.8/1,000, respectively (Fig 1D). Finally, the numbers of states with any programs were 5 in 2003-2004, 20 in 2013-2014, and 12 in 2021-2022 (Fig 2).

In summary, after a peak in the 2010s, the numbers of dialysis PCT training programs at US postsecondary institutions and program graduates (who have been primarily female and of minoritized race/ethnicity) have steadily declined. Programs offered by for-profit schools disproportionately declined, likely related to school closures.^5^^,^^6^ Even at their peak, the programs were graduating numbers of students that represented ∼2% of the existing US PCT workforce; graduates could have filled only ∼6% of open PCT FTEs in 2021-2022, suggesting that most PCTs are trained by other types of programs. For example, of the 642 training programs listed by California’s Department of Public Health in 2023-2024, most were associated with employers, and only 1 was a postsecondary program.^3^ Most US states have not had any postsecondary training programs for the past 20 years, and the programs remain geographically sparse. Within states, the number of programs was often uncorrelated with potential state needs for dialysis PCTs.

Although we cannot assess whether postsecondary programs provide better training than the employer programs used by most dialysis PCTs, the availability of more postsecondary training programs for these essential frontline workers might enable the standardization of training for US dialysis PCTs, in alignment with the training for respiratory therapists, who are better integrated into their healthcare teams than dialysis PCTs.^7^ Given that financial considerations are likely strong drivers of the decision to train via employer programs, the use of standardized curricula (supported by PCTs) at postsecondary training programs could potentially be endorsed by the National Association of Nephrology Technicians/Technologists and/or incentivized by policies such as employer tuition reimbursement.^8^^,^^9^ With the increasing number of patients receiving hemodialysis, increasing demands on dialysis providers, and growing need for home dialysis support, the need for well-trained PCTs continues to grow.^1^^,^^10^ Thus, the potential for the expansion, review, and standardization of training programs, at postsecondary institutions and elsewhere, should be explored.
